# Competition among the attentional networks due to resource reduction in Tibetan indigenous residents: evidence from event-related potentials

**DOI:** 10.1038/s41598-017-18886-7

**Published:** 2018-01-12

**Authors:** Delong Zhang, Xinjuan Zhang, Hailin Ma, Yan Wang, Huifang Ma, Ming Liu

**Affiliations:** 1 Center for the Study of Applied Psychology, Key Laboratory of Mental Health and Cognitive Science of Guangdong Province, School of Psychology, South China Normal University, Guangzhou, China; 2 Plateau Brain Science Research Center, South China Normal University/Tibet University, Guangzhou, 510631/Lhasa, 850012 China; 3 Institute for Brain Research and Rehabilitation, South China Normal University, Guangzhou, 510631 China; 4 Key Laboratory of Mental Health, Institute of Psychology, Chinese Academy of Sciences, Beijing, China; 5 College of Management, Tianjin University, Tianjin, China

## Abstract

This study used the attention network test (ANT) to evaluate the alerting, orienting, and executive network efficiencies of attention related to indigenous residents who were born and raised until early adulthood in different high-altitude areas (2900-m, 3700-m, and 4200-m) at the same location (3700-m) where these residents had been living for approximately 2 years in Tibet. We further applied the event-related potential (ERP) method to identify the underlying neurophysiological basis. Based on the ANT, we found that, in the 4200-m residents, executive function was increased but the orienting function was decreased, and the executive and orienting network scores were oppositely correlated. The behavioral findings were supported by the ERP data, showing that the P3 amplitude changes indicated that the executive function was over-active under conflict conditions and that the N1 amplitude change indicated a decreased orienting function in the 4200-m residents. In addition, the changed P3 amplitudes were significantly correlated with intelligence performance across the residents only in the 4200-m group. The present study provided evidence for competition among the attentional networks due to high-altitude exposure in indigenous residents, and showed the existence of a threshold of the influence of high altitudes on attentional function in the brain.

## Introduction

Previous studies have indicated that high-altitude exposure influences human attentional abilities^1^. The finding that attentional function changes as a result of high-altitude exposure was observed in behavioral performances^2–4^. In addition, the changed attentional function due to high-altitude exposure may contribute to the decreased attentional resources^5^. It should be noted that the although knowledge about changes in attentional function due to high-altitude exposure are mostly derived from acute^6,7^ or long-term^5^ high-altitude exposure in immigrants, little is known about the changes in attentional function due to high-altitude exposure in indigenous residents who have adapted to a high-altitude environment for generations.

Attentional function is fundamental and critical for performance of goal-directed behaviors, which guide the allocation of cognitive resources in response to changing environmental demands^8,9^. Attention is not a single unified system but comprises separate yet interrelated anatomical and functional networks responsible for the alerting, orienting, and executive control^5^. Of these attentional networks, the alerting network governs the capacity to sustain an alert state^10^, the orienting network regulates the focused identification and selection of sensory stimuli to covertly direct attention^11^, and the executive control network orchestrates the capacity of response monitoring of conflicts associated with a goal or inhibition of a contextually inappropriate response^12,13^. Previous studies have shown the existence of a certain degree of independence between the attentional networks^13^, but there are also obvious interactions between them^14^. Nevertheless, the attention network test (ANT)^13^, which combines the spatial cuing task^15^ and the Eriksen Flanker task^16^, can effectively measure these different attentional network efficiencies. Based on this approach, many previous studies have shown the factors influencing attentional function, including disease (Alzheimer^17^; Multiple sclerosis^18^; Schizophrenia^19^; Mild Cognitive Impairment^20^), aging^21^, and training^22^. Moreover, the ANT combined with electrophysiological methodology has been used to characterize the three attentional networks^21,23^. Indeed, the ERP approach provided an avenue to show differential effects of the attentional networks at various scalp locations, in which the posterior P1 and N1 components were used to represent early visual processing of stimulus properties^23^, and the midline P3 component serving as a marker of an evaluation of possible outcomes^24^ and response inhibition^25^. Using the ERP approach, previous studies using the flank task^26^ and Go/Nogo task^27^ in immigrants with long-term high-altitude exposure who were born and spent until early adulthood in low-altitude areas have suggested that high-altitude exposure clearly influences the response-conflict evaluation ability and the response-inhibit performance related to executive control function. It should be noted that cognition impairments in the immigrants may be different than those of indigenous residents. To date, the cognitive changes that are due to high-altitude exposure are still controversial in the high-altitude indigenous residents^28,29^, in which it should be that the high-altitude indigenous residents may adapt to high-altitude environments within a threshold of approximately 4000 m^28^.

To systematically explore the changes in attentional function due to high-altitude exposure in indigenous residents, the present study applied the ANT paradigm to measure the attention of young indigenous people who were born and raised at three different high altitudes (3000 m, 3700 m, and 4200 m) in Tibet. In addition, we employed the amplitudes of the N1 and P1 components to measure the alerting and orienting functions of attention related to the three altitudes, as well as the amplitude of the P3 component in relation to executive control function.

## Results

### Behavioral results

The mean reaction times (RTs) and mean accuracy of each experimental condition related to the three groups are summarized in Table 1.

**Table 1:** Behavioral performance of the experimental conditions related to the three groups.

Group	No cue		Spatial cue		Center cue	
	Incongruent	Congruent	Incongruent	Congruent	Incongruent	Congruent
Mean RTs (ms) and standard deviations						
**2900 m**	665 (12.8)	581 (11.1)	590 (14.4)	533 (12.1)	656 (11.3)	567 (12.1)
**3700 m**	741 (17.4)	648 (12.3)	654 (17.4)	597 (13.9)	728 (15.9)	641 (13.2)
**4200 m**	697 (20.4)	653 (13.8)	669 (17.6)	625 (15.9)	691 (19.2)	645 (12.5)
Accuracy (%) and standard deviations						
**2900 m**	95.7 (0.008)	99.6 (0.001)	97.1 (0.007)	99.2 (0.020)	93.9 (0.011)	99.0 (0.003)
**3700 m**	95.6 (0.009)	98.2 (0.004)	95.6 (0.009)	98.8 (0.040)	95.5 (0.010)	98.6 (0.004)
**4200 m**	97.8 (0.005)	98.7 (0.004)	98.8 (0.003)	98.8 (0.030)	97.8 (0.005)	98.7 (0.003)

### Alerting effect

With respect to the alerting network, a one-way ANOVA with altitude as a factor did not indicate a significant effect of altitude (F (2, 64) = 0.237, *p* = 0.790).

### Orienting effect

The orienting network scores were also analyzed using a one-way ANOVA and suggested that there was a significant main effect of altitude [F (2, 64) = 8.618, *p* = 0.001]. Further analysis showed that the orienting scores of the 4200-m altitude participants were significantly less than those of the 2900-m altitude participants (LSD, *p* < 0.05). In addition, the orienting scores of the 4200-m altitude participants were significantly less than those of the 3700-m altitude participants (LSD, *p* < 0.05), but there was no significant difference in the orienting scores between the 2900-m and 3700-m altitude groups (LSD, *p* > 0.05).

### Executive effect

Using a one-way ANOVA, we found that there was a significant main effect of altitude on the executive network scores [F (2, 64) = 4.384, *p* = 0.014]. A post hoc analysis found that the executive scores of the 4200-m altitude residents were smaller than those of the 3700-m altitude residents and those of the 2900-m altitude residents (LSD, *p* < 0.05). Of note, there was no significant difference in the executive scores between the 3700-m and 2900-m altitude residents (LSD, *p* > 0.05).

The results of the attention network scores are summarized in Table 2.

**Table 2:** Attentional network scores based on the RTs of the three groups (*M* ± *SD*).

	2900 m	3700 m	4200 m	*F*
Alerting	9.17 ± 2.75	7.67 ± 4.50	5.91 ± 2.88	0.237
Orienting	52.00 ± 4.42	60.38 ± 5.54	22.39 ± 9.26	8.618***
Executive	76.87 ± 6.90	79.29 ± 5.44	44.52 ± 13.16	4.384*

Note: *p < 0.05; ***p < 0.001.

### ERP results

**Figure 1:**
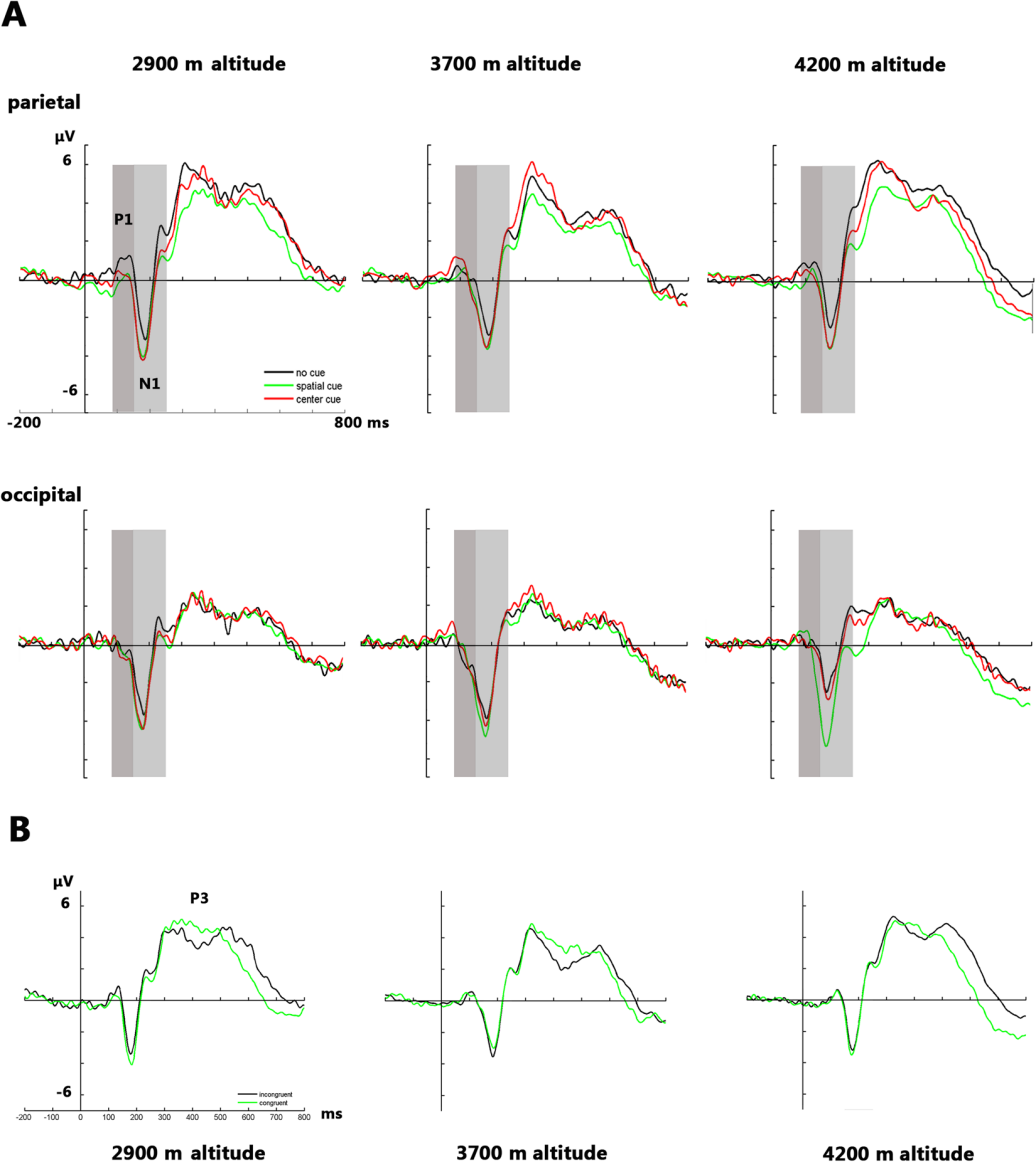
ERP waveforms of the three altitude groups. The ERP waveforms were located at the posterior sites, and, by averaging within groups, they showed the main effect of each cue condition (**A**). The ERP waveforms were located at the posterior sites, and by averaging within groups they showed the main effect of the conflict condition (**B**).

**Figure 2:**
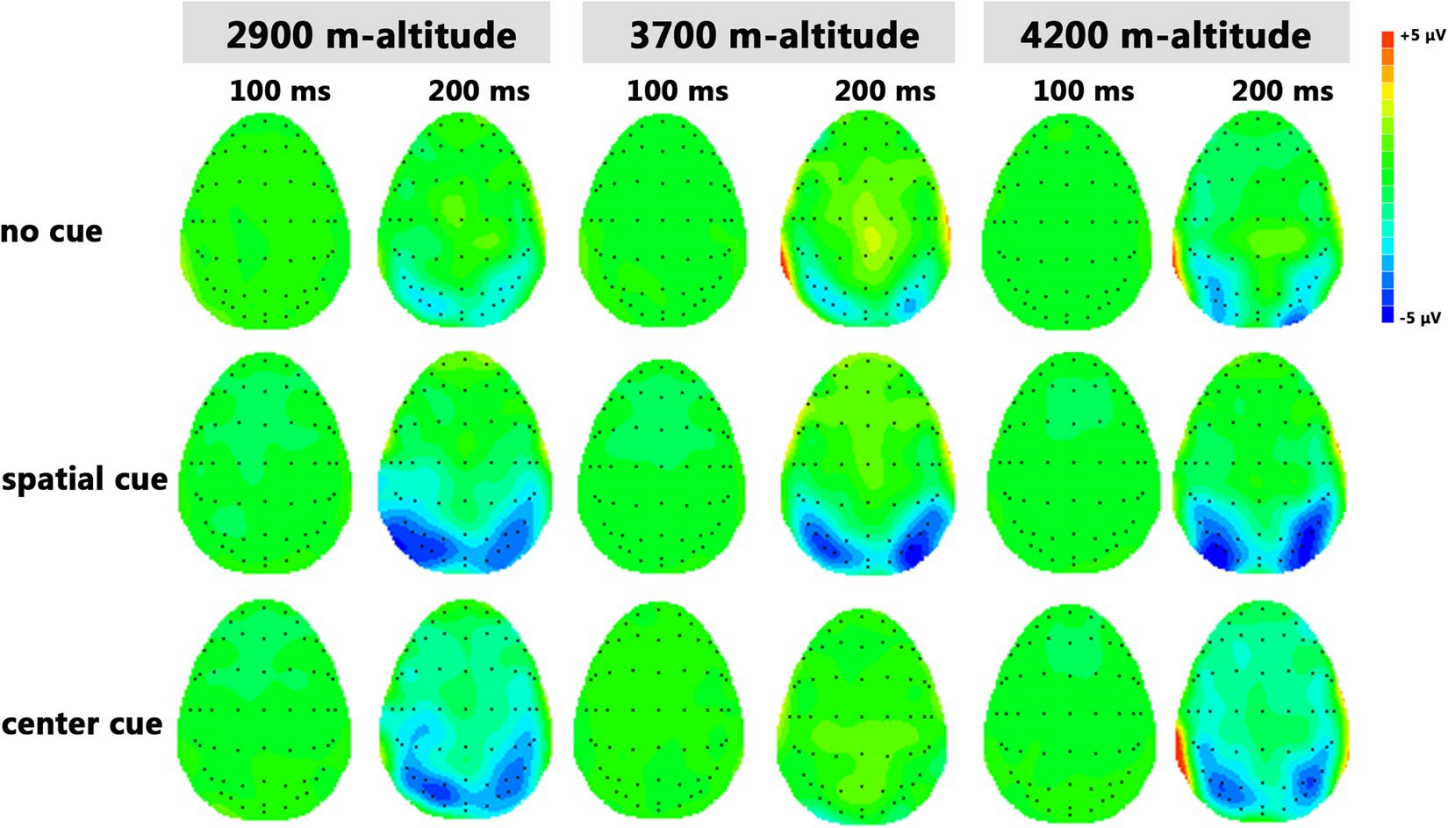
Topographic map of the EEG activity by the main effect of the cue condition following target presentation in the three groups.

**Figure 3:**
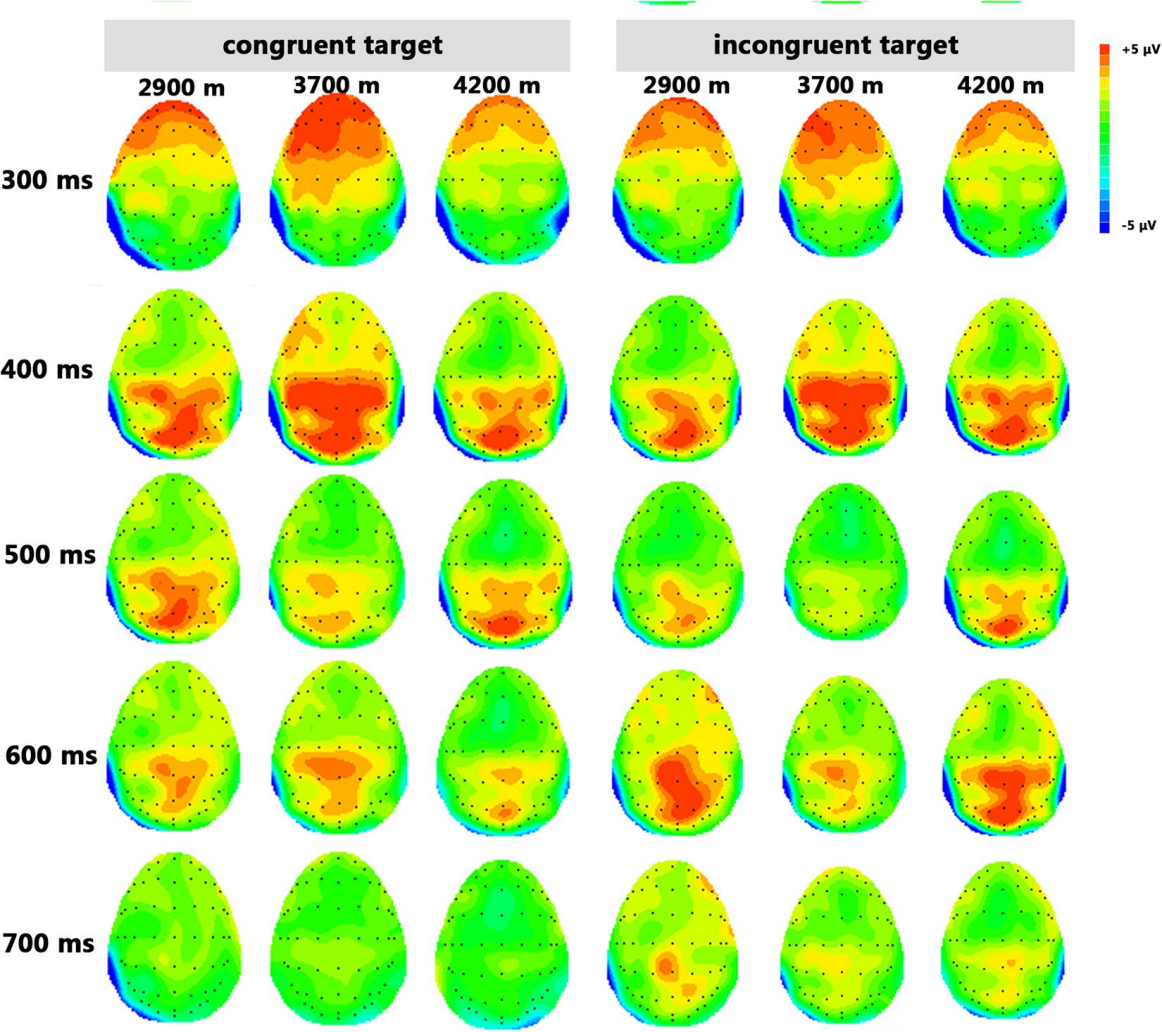
Topographic map of the EEG activity by the main effect of the conflict condition following target presentation in the three groups.

The ERP waveforms are shown in Fig. 1, and the topographic maps are shown in Figs 2 and 3.

### Alerting

#### Posterior target N1 amplitude

With respect to the normalized target N1 amplitude, the main effect of the brain area was significant (F (1, 64) = 35.498, *p* = 0.001), showing that the N1 amplitude of the occipital area (0.618 ± 0.14 μV) was more negative than that of the parietal area (1.133 ± 0.16 μV). Neither the main effect of hemisphere (F (1, 64) = 0.389, *p* = 0.678) nor that of altitude (F (1, 64) = 0.771, *p* = 0.467) was significant. The interaction between altitude and brain area was not significant (F (1, 64) = 0.284, *p* = 0.754), and no significant interaction was observed between altitude and hemisphere (F (1, 64) = 2.079, *p* = 0.102).

#### Posterior target P1 amplitude

With respect to the normalized target P1 amplitude, the main effect of brain area was significant (F (1, 64) = 16.849, *p* = 0.001), indicating that the P1 amplitude of the parietal area (0.281 ± 0.13 μV) was more positive than that of the occipital area (−0.059 ± 0.12 μV). The main effect of hemisphere was not significant (F (1, 64) = 0.108, *p* = 0.875), and also, the main effect of altitude was not significant (F (1, 64) = 1.378, *p* = 0.260). The interaction between altitude and brain area was not significant (F (1, 64) = 2.439, *p* = 0.095), and there was no significant interaction between altitude and hemisphere (F (1, 64) = 0.887, *p* = 0.464).

### Orienting

#### Posterior target N1 amplitude

With respect to the normalized target N1 amplitude, the main effect of altitude was significant (F (2, 64) = 3.579, *p* = 0.034); we found that the N1 amplitude of the 4200-m altitude group (−0.367 ± 0.13 μV) was more negative than that of the 2900-m altitude group (0.130 ± 0.13 μV) and that of the 3700-m altitude group (−0.020 ± 0.14 μV). The main effect of brain area was significant (F (1, 64) = 23.222, *p* = 0.001), in which the N1 amplitude of the occipital area (−0.254 ± 0.08 μV) was more negative than that of the parietal area (0.082 ± 0.09 μV). The main effect of hemisphere was not significant (F (1, 64) = 1.975, *p* = 0.143). The interaction between altitude and brain area was not significant (F (1, 64) = 0.445, *p* = 0.643), and no significant interaction was found between altitude and hemisphere (F (1, 64) = 0.621, *p* = 0.638).

#### Posterior target P1 amplitude

Regarding the normalized target P1 amplitude, the main effect of brain area was significant (F (1, 64) = 38.783, *p* = 0.001), indicating that the P1 amplitude of the occipital area (−0.025 ± 0.07 μV) was more positive than that of the parietal area (−0.358 ± 0.08 μV). The main effect of hemisphere was significant (F (2, 63) = 8.295, *p* = 0.001); we found that the P1 amplitude of the left hemisphere (−0.151 ± 0.71 μV) was more positive than that of the middle hemisphere (−0.330 ± 0.08 μV), as was the P1 amplitude of the right hemisphere (−0.093 ± 0.08 μV). In addition, the interaction between brain area and hemisphere was significant (F (1, 64) = 22.754, *p = *0.001); in the parietal area, the P1 amplitude of the left hemisphere (−0.294 ± 0.08 μV) and that of the right hemisphere (−0.165 ± 0.08 μV) were both more positive than that of the middle hemisphere (−0.615 ± 0.11 μV). The main effect of altitude was not significant (F (1, 64) = 1.175, *p* = 0.315). The interaction between altitude and brain area was not significant (F (1, 64) = 1.534, *p* = 0.224), and there was no significant interaction between altitude and hemisphere (F (1, 64) = 0.334, *p* = 0.810).

### Executive

#### Target P3 amplitude

Regarding the aspect of the normalized target P3 amplitude, the main effect of altitude was significant (F (2, 64) = 3.036, *p* = 0.055), suggesting that the P3 amplitude of the 4200-m altitude group (0.727 ± 0.23 μV) was more positive than that of the 3700-m altitude group (−0.068 ± 0.24 μV). The main effect of the brain area was significant (F (1, 64) = 5.826, *p* = 0.009), showing that the P3 amplitude of the CPZ (0.614 ± 0.26 μV) and that of the PZ (0.563 ± 0.13 μV) were more positive than those of the FCZ(−0.080 ± 0.14 μV) and the CZ (0.153 ± 0.15 μV). There was no significant interaction between altitude and brain area (F (1, 64) = 0.406, *p* = 0.740).

### Correlation analyses

**Figure 4:**
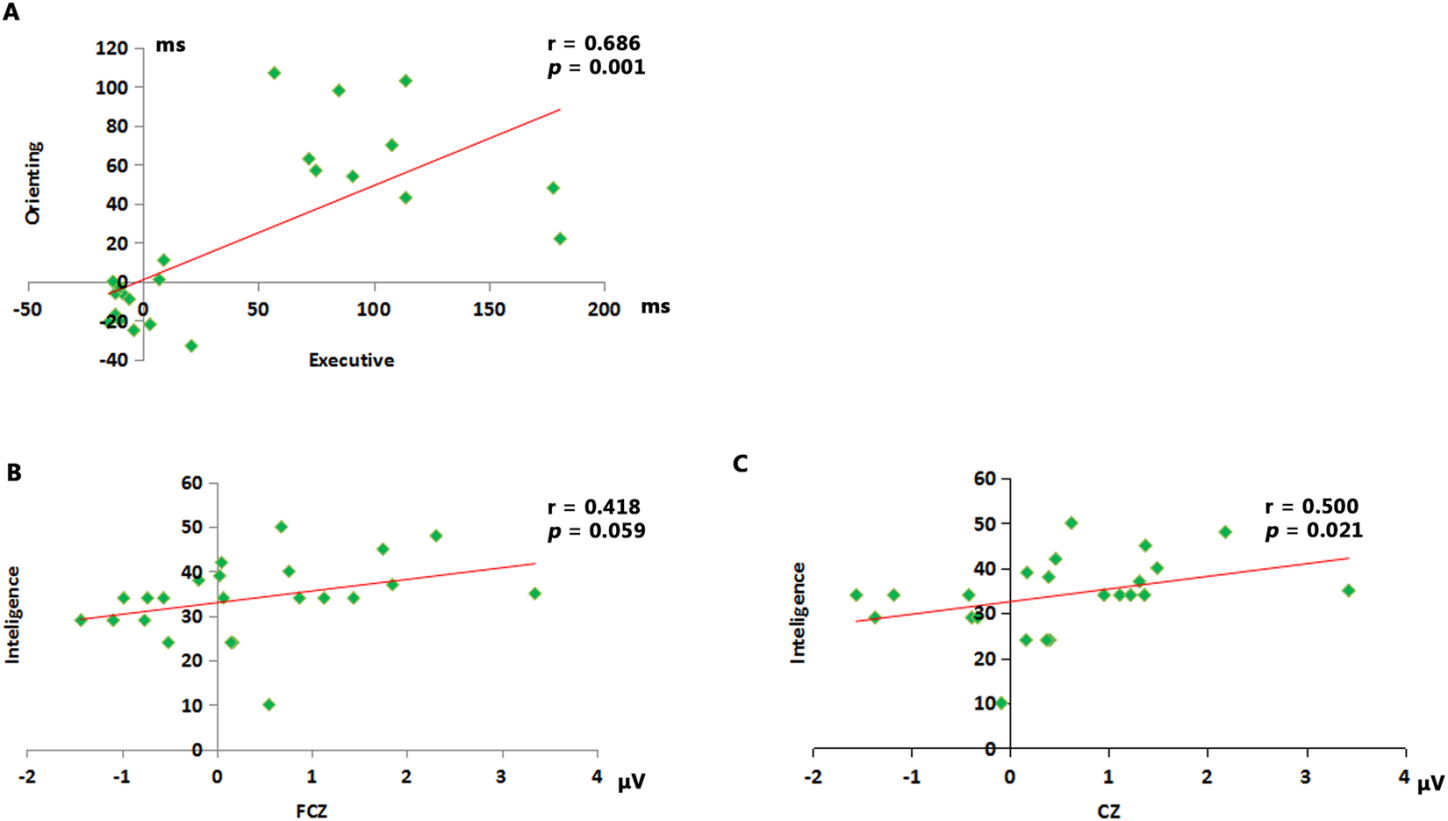
The correlation results within the 4200 m altitude group.

Table 3 shows the correlations among the scores of the three attentional networks related to the three groups. We found that the orienting score was positively correlated with the executive score in the 4200-m altitude group (*p* < 0.01) (Fig. 4A). There were no significant correlations among the three attentional network scores within the other two groups.

**Table 3:** Correlation coefficients between the attentional networks in the three groups.

	2900 m			3700 m			4200 m		
	A	O	E	A	O	E	A	O	E
Alerting	1	—	—	1	—	—	1	—	—
Orienting	0.015	1	—	−0.014	1	—	−0.051	1	—
Executive	−0.048	−0.099	1	0.177	−0.306	1	0.030	0.686**	1

Note: **p < 0.01. A, alerting; O, orienting; E, executive.

Table 4 shows the correlations between the executive component (the normalized amplitude of P3) and intelligence within the three groups. Only in the 4200-m altitude group did the intelligence score exhibit a positive correlation with the P3 normalized amplitude at the FCZ (Fig. 4B) and the CZ (Fig. 4C) sites.

**Table 4:** Correction between the normalized P3 amplitude and the intelligence score within the three groups.

Altitude	2900 m		3700 m		4200 m	
Components	*r*	*p*	*r*	*p*	*r*	*p*
FCZ-P3	−0.206	0.370	0.033	0.893	0.418*	0.059
CZ- P3	−0.232	0.312	0.181	0.458	0.500*	0.021

Note: *p < 0.05.

## Discussion

The present study aimed to explore the attentional network efficiency of high-altitude indigenous residents in Tibet via combining the ANT and ERP approaches. The main findings can be summarized as follows: 1) the 4200-m altitude indigenous residents specifically exhibited over-active executive control function responding to the conflict condition in behavioral performances, and the enhanced degree of executive control function was oppositely associated with the change in orienting function; 2) the neurophysiology marks (i.e., the P3, P1, and N1) related to attentional function correspondingly supported the behavioral performances across the three high-altitude groups; 3) the P3 amplitude alterations related to the executive control function in the 4200-m altitude indigenous residents was significantly associated with their intelligence performances. All these observations in the 4200-m altitude residents were not found in either the 3700-m or in the 2900-m altitude residents.

Many previous studies have shown the existence of independent brain attentional networks related to the alerting^10^, orienting^30^, and executive systems^13^. The efficiencies of these attentional networks can be quantitatively measured using the ANT approach^13^. By using the identical ANT experimental setting as the present study, Fan and his colleagues measured the efficiencies of the attentional networks in sea-level residents, and determined the independence of the executive, orienting, and alerting attentional functions. By using the same approach, the present study measured the influence of alerting, orienting, and executive functions of high-altitude indigenous residents on behavioral performances. On the one hand, the present study determined the independence among the attentional networks in the 2900-m and 3700-m residents just as the previous study^13^. On the other hand, the present study first indicated the significantly negative association between the executive and the orienting scores in the 4200-m high-altitude residents. When compared with the residents of the 2900-m and 3700-m groups, we found that the executive network scores were significantly decreased in the 4200-m altitude group. According to the definition of the executive network score, a smaller executive score indicates better executive control efficiency^31,32^. The results of the present study provided evidence for the increased executive function in the residents who were born and lived until early adulthood in the 4200-m altitude areas compared with those in the 2900-m and 3700-m altitude areas. Meanwhile, the present findings also found a decreased orienting network score in the 4200-m altitude resident group compared with those of the other two groups. It should be noted that a smaller orienting score indicates worse efficiency of the orienting function based on the definition of the orienting score^31,32^. Moreover, this study indicated that the executive score was positively correlated with the orienting score in the 4200-m altitude residents, showing that the increased executive function was significantly associated with the decreased orienting function in these residents.

The important issue that the data of the present study were only derived from the high-altitude areas considering the well-controlled influence of the genetic and living culture and something such as this, should be kept in mind. The data of the present study did not provide knowledge about the change in the attentional networks of the high-altitude residents related to the sea-level residents. Based on this consideration, the observed changes in the attentional networks across the three high-altitude groups could not provide indications about the attentional network alterations from normal attentional function at sea-level. Instead, the alterations in the attentional networks across the three high-altitudes groups could only exhibit the influence of high altitudes on attentional function. Another important issue that should also be noted is that the divergence of the present participants relied on the high altitude of the areas where they had been born and living until early adulthood instead of the high altitude of the locations where they were currently living, since all of these participants had been living in the same city for about two years. Regarding this aspect, the findings of the present study demonstrated the impact of high-altitude exposure on attentional function during the early growth period rather than on the adaption to the high-altitude environment.

As mentioned above, previous studies have stated that attentional networks are independent in normal brain function^13^, since the executive, orienting, and alerting functions may be derived from attentional sources that are supported by different attentional systems within distinct anatomical areas^25^. Despite this, many previous studies have still shown a reduction in the dissociation among these attentional networks^14^. For instance, a previous study using the ANT approach indicated the selective impairments in executive function and showed the interaction between the executive and orienting function in AD patients^33^. To provide more experimental evidence for the existence of less differentiation among the attentional networks, the present findings showed that the executive and orienting functions were significantly correlated with each other in the 4200-m residents. Of course, the less differentiation of the executive and orienting functions was not only observed in the specific population of the 4200-m residents in the present study but also in another specific population, namely, preterm children^34^. A previous study reported that a stronger relation between the orienting and executive control networks was found in pre-term children than that in full-term children, suggesting the existence of less differentiation between the two networks in the preterm children^34^. More possibly, the data of the present study might support that there is a similarity in the attentional function between preterm children and the residents who were born and raised until early adulthood within the 4200-m altitude areas.

In addition to the behavioral performance measurement based on the ANT paradigm, the present study further applied the ERP components to underpin the behavioral observations. First, we employed the P3 amplitudes as an index to identify the neurophysiological basis underlying the executive function of the high-altitude residents. As we know, the P3 amplitude is elicited by numerous executive control tasks^35^, and a conflict condition usually leads to an increased P3 amplitude^25^, in which the increased amplitude may be related to the enhanced response inhibition^25^. In this study, we found that the target P3 amplitude was more positive in the incongruent condition than that in the congruent condition, which further showed that there was an increased response inhibition to the conflict context. More importantly, we found that the distance value of the P3 amplitude between the incongruent and the congruent conditions was positively correlated with the intelligence performance in the 4200-m altitude residents only. The observation above showed that the alteration degree of the P3 amplitude between the incongruent and the congruent conditions was highly correlated with intelligence, providing experimental evidence for the tight association between executive control ability and intelligence. Indeed, as many previous studies have already proposed that the tradeoff between accuracy and processing speed based on the modulation of the executive control function is the core element of intelligence^36^, the present findings provided further experimental evidence for the important role of executive control function in intelligence and that the core role of executive control function in intelligence was more obvious in the 4200-m altitude residents.

Regarding the aspect of attentional network efficiency, this study indicated a decreased orienting function of attention in the 4200-m residents. Corresponding to this finding, we found that the target N1 amplitude of the 4200-m residents was more negative than those of the other groups of residents, as we know that the N1 component is an early stage component whose amplitude is enhanced in conditions of heightened attention; thus, we used this component as a marker of early visual processing of stimulus properties^37,38^. The data of the present study showed that the mental resource was limited in the 4200-m residents during the orienting processing compared with that of the 2900-m residents and that of the 3700-m residents. Of note, the observed mental resource was decreased in the 4200-m residents, a finding that highly corresponds with the findings in terms of the behavioral performance indicating that the orienting function had decreased. Regarding this aspect, the findings of the present study also showed the deficit of attentional modulation of the orienting function in the 4200-m residents. In addition, the change in the N1 component clearly supported the findings of the behavioral performance.

When compared with the 3700-m and 2900-m residents, the executive and orienting attentional functions were specifically altered in the 4200-m residents. Both the behavioral performances and the ERP component changes showed opposite responses between the executive and orienting functions in the 4200-m residents. More importantly, the degrees of change between the executive and orienting scores were consistent and statistically significant. That is, the extent of improvement in executive function significantly corresponded with the degree of decrease in the orienting function of the 4200-m residents. In addition, these observations in the 4200-m residents were not found in the other two groups, suggesting the existence of a specific brain functional state only in the 4200-m residents. The most possible interpretation of these findings above may be that the reduction of the attentional resource in the 4200-m residents induced a significant competition among the brain attentional networks. Of course, there are still few studies focusing on this issue, which limited the speculation of the present study; therefore, more future studies to extend the experimental evidence on this question are needed.

The most important contribution of the present study may be that the present study is the first that focused on indigenous residents who were born and raised until early adulthood at different high-altitude areas in Tibet. We found that the altitude degree where the indigenous residents grew up exhibited a significant impact on attentional function, especially on the executive and orienting functions, in which the majority of the changes were observed in the 4200-m indigenous residents.

In conclusion, the present study combined the ANT paradigm and the ERP technique to explore changes in the attentional networks of indigenous residents related to different high-altitude areas in Tibet. We found that growing up in areas above 4200 m significantly induced an over-active executive function but decreased the orienting function of the attentional network, which was not observed in the 3700-m and the 2900-m residents. The findings of the present study might, on the one hand, support the existence of competition among the attentional networks due to high-altitude exposure in indigenous residents and, on the other hand, might support the existence of a threshold of the high-altitude influence on brain attentional function.

## Methods

### Participants

We recruited a total of seventy-five right-handed, healthy high-altitude residents (aged 18–23 years) from the campus of Tibet University for the present study. These residents belonged to three groups according to the altitudes of the locations where they were born and raised until early adulthood (i.e., Linzhi: 2900 m, Shannan/Rikaze: 3700 m, and Naqu/Ali: 4200 m). Of these residents, 25 were collected for each group (i.e., *n* = 25 × 3). Although these participants were born raised until early adulthood at different altitudes, all of them had moved to Lhasa (3700 m) for studying at Tibet University for the two years prior to the study. All these participants had normal or corrected-to-normal vision. None of them had a history of neurological or psychiatric disorders. Eight participants were excluded because of their low accuracy rate, frequent eye moments, and/or excessive artifacts in the electroencephalogram (EEG), ultimately leaving 23 participants in the 2900-m altitude group, 21 participants in the 3700-m altitude group, and 23 participants in the 4200-m altitude group. The three groups were matched in respect to age, gender, education, and intelligence scores (Raven Progress Matrices) (all *p* > 0.05) (Table 5). This study was approved by the Ethics Committee of the Institute of Psychology, Chinese Academy of Sciences and was conducted according to the Declaration of Helsinki. We obtained written informed consent from each participant before the experiment.

**Table 5:** Age, gender, education, immigrant time, and intelligence scores (Raven Progress Matrices) of each group.

	2900 m	3700 m	4200 m	*p* value
Age (yrs)	20.91 ± 1.24	21.29 ± 0.90	20.87 ± 1.39	0.462
Gender (M/F)	12/11	11/10	12/11	1.000
Education (yrs)	14.04 ± 0.97	14.38 ± 0.59	14.13 ± 0.81	0.372
Immigrant time (yrs)	2.04 ± 0.97	2.38 ± 0.59	2.13 ± 0.82	0.372
Intelligence	36.08 ± 8.20	32.095 ± 8.22	33.96 ± 8.73	0.294

**Note**: M, male; F, female; immigrant time refers to the time they had lived in Lhasa.

### Attention network test (ANT)

**Figure 5:**
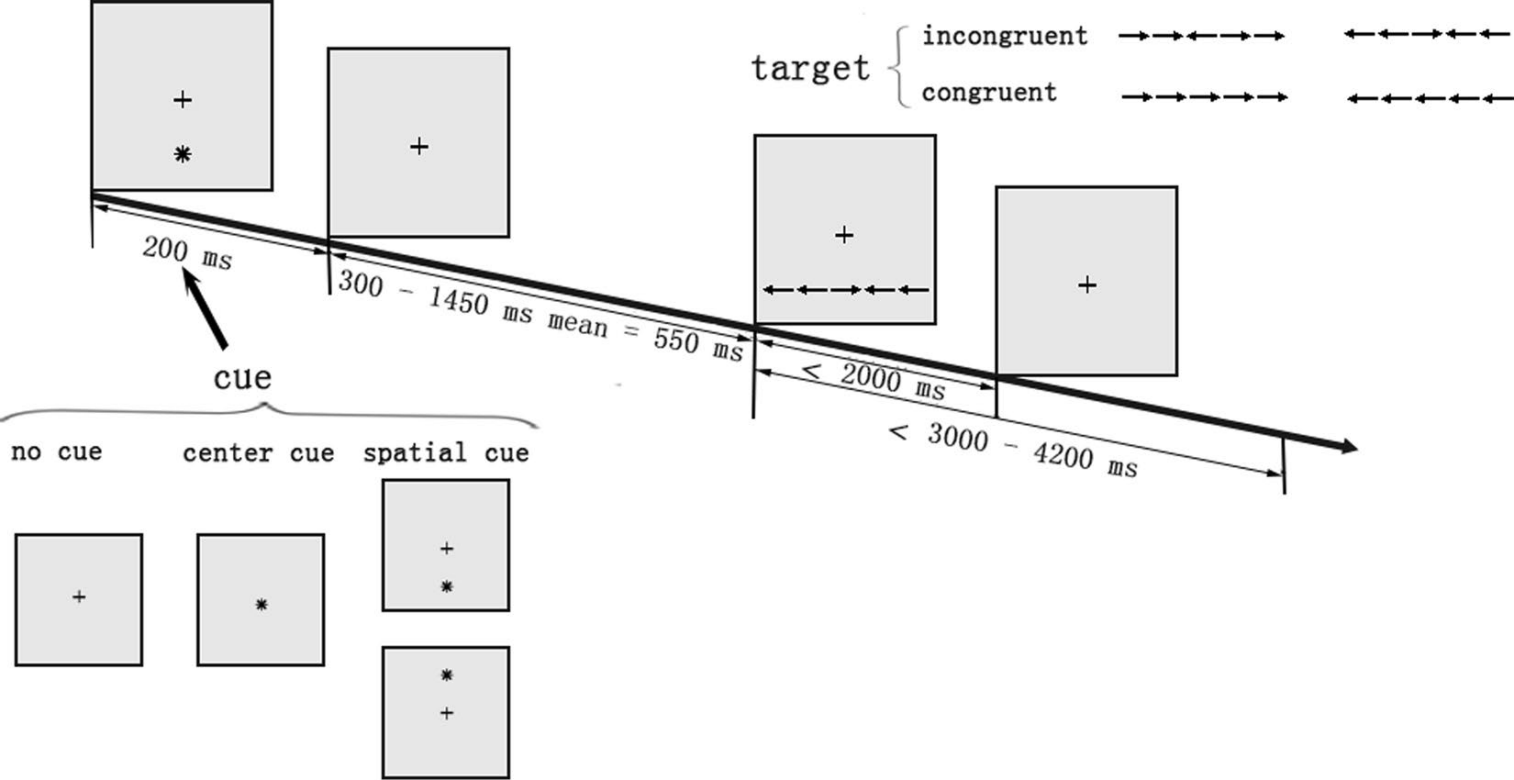
Schematic of the ANT procedure.

The ANT is used to explore the efficiency of the three specialized attentional networks (i.e., alerting, orienting, and executive-control)^32^. The schematic procedure of the ANT is shown in Fig. 5. Briefly, the ANT includes three cue conditions (i.e., no cue, center cue, and spatial cue) and two target conditions (i.e., congruent and incongruent). During congruent trials, the arrowheads are pointed in the same direction, to the opposite direction during the incongruent trials. A cue is presented before the target appearance. All stimuli appeared in black against a gray background. Each arrow subtended 0.58° of the visual angle and was separated from neighboring arrows by 0.06° of the visual angle. The stimuli (one center arrow and four flanker arrows) subtended a total 3.27° of the visual angle. The row of five arrows was presented in one of two locations, 1.06° either above or below the fixation cross. The trials were presented using a block design, and each block had 6 conditions (i.e., two target types and three cues) in equal proportions. A total of 108 trials presented in 6 blocks were included. Participants were instructed to respond to the direction of a central arrow; “F” corresponded to the central arrow pointing to left, and “J” to the central arrow pointing to right. A complete practice was performed before the experiment.

This study employed the *E*-prime software system (Version 2.0, Psychology Software Tools, Inc., Pittsburgh, PA) to complement the stimuli presentation and behavioral data collection. The scores of the three attentional network scores were defined based on the RTs as follows: alerting effect = RT_no cue_ − RT_cente rcue_, orienting effect = RT_center cue_ − RT_spatial cue_, and conflict effect = RT_incongruent_ − RT_congruent_. In addition, the ERP components were normalized according to the definition of the attentional network scores as follows: alerting effect = component_no cue_ – component_center cue_, orienting effect = component_center cue_ − component_spatial cue_, and conflict effect = component_incongruent_ − component_congruent_.

### ERP recording

This study recorded EEG data using 64 scalp sites (10/20 system) with Ag/AgCl electrodes mounted in an elastic cap in CURRY 7 (http://compumedicsneuroscan.com/products-overview/). The EEG data were referenced in the right mastoid (M2), and using a ground electrode on the medial frontal aspect. A vertical electrooculogram (VEOG) was recorded with electrodes that were placed above and below the left eye. A horizontal electrooculogram (HEOG) was monitored using two electrodes 10 mm from the outer canthi of both eyes. All inter-electrode impedances were maintained below 5 kΩ, and the EEG signals were 0.05–100 Hz bandpass and digitized at 500 Hz.

### Data analysis

#### Behavioral data analysis

The average RTs of each of the six experimental condition combinations (i.e., the three cuing conditions by two target conditions) were calculated. Then, the three attentional network scores were calculated based on the operational definitions. Notably, to calculate the alerting and orienting network scores, the mean RTs from the two target conditions under each of the three cue conditions were averaged. In addition, for the calculation of the executive-control score, the mean RTs from the three cue conditions were averaged for the two target conditions. Of note, the incorrect responses were not included in the RT analysis, and the extreme RT of trials within each condition (mean ± 3 standard deviation) were removed for the calculation in each subject. One-way ANOVA was carried out for the RTs according to the definition of attentional networks.

#### ERP data analysis

The initial off-line processing of the EEG data was complemented by the CURRY 7 Neuroimaging Suite (Compumedics USA, Inc., Charlotte, NC, USA). The EEG date were rereferenced to the average of the left and right mastoids, and digitally filtered with a 40 Hz low-pass filter. Trials with eye-blink/eye-movements or muscle activity were excluded from further analysis. The independent component analysis (ICA) method was used to identify vertical and horizontal ocular artifacts, and this study further removed them from the signal, trials with various artifacts were rejected with a criterion of ±75μV. Only the correct response trials were included for the average ERP calculations, thus excluding 4255 (9.8%) of the 43416 total trials (108 × 6 × 67)). ERPs for the correct trials were segmented into epochs that were time-locked according to the target onset. The target-related ERP component was further normalized according to the definition of the attention network test scores^32^. The data were divided into epochs of 1000 ms in length, including a 200 ms interval before the target onset. Each channel was baseline-corrected using the prestimulus 200 ms interval. The ERPs were averaged separately for the cue conditions and target conditions.

The posterior target P1/N1 mean amplitudes were analyzed at the time period 80–150 ms and 150–250 ms, respectively, following the presentation point of target stimuli. These components were averaged at the parietal (i.e., P3, Pz, and P4) and the occipital (i.e., O1, Oz, and O2) electrodes, respectively; this region of interest was selected based on previous studies^39^. A mixed-model ANOVA was applied to the different components. The ANOVA factors for the P1/N1 mean components included the brain areas (i.e., the occipital site and the parieto-occipital site) and the hemisphere (i.e., left, middle, right) as the within-subject factors, and the altitude (i.e., 2900 m, 3700 m, and 4200 m) as the between-subject factor for a 2 × 2 × 3 design. The P3 amplitude was considered to be related to the executive-control function. The P3 mean amplitude was examined at the time period of 300–800 ms following the presentation of target stimuli. Both amplitudes were calculated using the FCZ, CZ, CPZ, and PZ sites, this region of interest was selected based on previous studies^39^; a mixed-model ANOVA was applied to the P3 components including the brain areas (i.e., FCZ, CZ, CPZ, and PZ) as the within-subject factor and the altitude (i.e., 2900 m, 3700 m, and 4200 m) as the between-subject factor for a 2 × 3 design. Specifically, the ERP components were normalized according to the attentional network score definitions as follows: alerting effect (P1/N1) = component_no cue_ (P1/N1) − component_center cue_ (P1/N1); orienting effect (P1/N1) = component_center cue_ (P1/N1) − component_spatial cue_ (P1/N1); and conflict effect (P3) = component_incongruent_ (P3) − component_congruent_ (P3). The Greenhouse-Geisser correction approach was used to compensate for sphericity violations. Post hoc analyses were conducted to explore the interaction effects^40^.
